# Gut Microbiome Characteristics in feral and domesticated horses from different geographic locations

**DOI:** 10.1038/s42003-022-03116-2

**Published:** 2022-02-25

**Authors:** Li Ang, Gabriel Vinderola, Akihito Endo, Juha Kantanen, Chen Jingfeng, Ana Binetti, Patricia Burns, Shi Qingmiao, Ding Suying, Yu Zujiang, David Rios-Covian, Anastasia Mantziari, Shea Beasley, Carlos Gomez-Gallego, Miguel Gueimonde, Seppo Salminen

**Affiliations:** 1 Health Management Centre, The First Affiliated Hospital of Zhengzhou University, Zhengzhou, China; 2 Department of Henan Gene Hospital, The First Affiliated Hospital of Zhengzhou University, Zhengzhou, China; 3 Department of Infection Disease, The First Affiliated Hospital of Zhengzhou University, Zhengzhou, China; 4 Instituto de Lactología Industrial (INLAIN, UNL-CONICET), Facultad de Ingeniería Química, Universidad Nacional del Litoral, Santa Fe, Argentina; 5 Department of Food, Aroma and Cosmetic Chemistry, Tokyo University of Agriculture, Hokkaido, Japan; 6 Production Systems, Natural Resources Institute Finland, Jokioinen, Finland; 7 Department and Microbiology and Biochemistry of Dairy Products, Instituto de Productos Lácteos de Asturias (IPLA-CSIC), Asturias, Spain; 8 Functional Foods Forum, Faculty of Medicine, University of Turku, Turku, Finland; 9 Institute of Public Health and Nutrition, University of Eastern Finland, Kuopio, Finland

**Keywords:** Microbiome, Metagenomics

## Abstract

Domesticated horses live under different conditions compared with their extinct wild ancestors. While housed, medicated and kept on a restricted source of feed, the microbiota of domesticated horses is hypothesized to be altered. We assessed the fecal microbiome of 57 domestic and feral horses from different locations on three continents, observing geographical differences. A higher abundance of eukaryota (*p* < 0.05) and viruses (*p* < 0.05) and lower of archaea (*p* < 0.05) were found in feral animals when compared with domestic ones. The abundance of genes coding for microbe-produced enzymes involved in the metabolism of carbohydrates was significantly higher (*p* < 0.05) in feral animals regardless of the geographic origin. Differences in the fecal resistomes between both groups of animals were also noted. The domestic/captive horse microbiomes were enriched in genes conferring resistance to tetracycline, likely reflecting the use of this antibiotic in the management of these animals. Our data showed an impoverishment of the fecal microbiome in domestic horses with diet, antibiotic exposure and hygiene being likely drivers. The results offer a view of the intestinal microbiome of horses and the impact of domestication or captivity, which may uncover novel targets for modulating the microbiome of horses to enhance animal health and well-being.

## Abstract (web summary)

Li Ang et al. present an investigation of feral and domesticated horse gut microbiomes across three continents. Their results provide new insight into how changes in horse lifestyle are reflected in the resident gut microbiome.

## Introduction

Intestinal microorganisms in both humans and animals have co-evolved with their host reaching a symbiotic relationship. The intestinal microbiome carries multiple genes for host health and survival, enabling optimizing energy harvested from food and subsequent rapid storage in the body fat tissue^1^. Other genes facilitate the production of vitamins and cofactors or produce biologically active microbe-associated molecules, such as short-chain fatty acids, indoles, tryptamine, peptidoglycan or lipopolysaccharide, which are important to health^2–4^.

Modern hygienic and antibiotic-dependent lifestyles have been related to a decline in microbiota abundance and diversity, and connects relationship of this depleted microbiome with an increased risk of chronic diseases has been hypothesized^5^. This dysbiosis of the microbiota is not exclusive to humans, and horses (*Equus ferus caballus*) are not an exception, with some dysbiotic states already reported in these animals^6, 7^. Horses were domesticated approximately 5500 years ago from their wild ancestors^8^, which have evolved from ancient times with an intestinal microbiome fitted to their environmental conditions; however, the changes related to domestication may have modified the microbiome, reducing resistance to diseases. Indeed, domestic horses are commonly known to be vulnerable to diseases originating in the gastrointestinal tract, where the microbiota reside and which is prone to disturbances and malfunctions^9^.

In humans, the microbiota of individuals not exposed to modern life was found to harbor a much higher microbial diversity than the microbiota of those living in Westernized societies^10, 11^. Similarly, the microbiota of domesticated animals, such as gayal or deer, has been found to be different from that of their wild-living counterparts^12, 13^. Microbiota changes related to domestication have been suggested to be common among mammals^14^. Therefore, it is likely that the ancient horse microbiota was richer and more resilient, conferring protection to environmental challenges, but modern veterinary practices and lifestyle may have changed it.

Studies on horse microbiome are still limited and the information available so far relies mainly in the use of 16S rRNA gene based sequencing strategies^6, 15–22^, with large metagenome studies still lacking. Furthermore, the impact of the modern management and feeding practices associated with domestication and housing and the comparison between domestic and wild-living subjects for horse microbiota have been addressed in only a few studies, which have shown some differences^23–25^. Therefore, further studies in this area are clearly needed. Preventing microbiome disturbances may help to maintain horse health by preventing and reducing the risk of diseases of intestinal origin, such as colitis, laminitis and metabolic diseases^26–28^.

The comparison of the fecal microbiome from horses in different geographical locations enables the characterization of the core horse microbiome and the understanding on the impact of local diets and conditions. This comparison also facilitates the assessment of the genes involved in the metabolism of dietary compounds as well as the potential differences in health-relevant genes, such as those involved in resistance to antibiotics. Domestic horses show an increasing occurrence of gastrointestinal and metabolic disorders, with colitis, grass sickness and laminitis being among the most common diseases^29^. In domestic environments, these conditions are treated commonly with medication and antibiotics^30, 31^. The use of medication is likely to affect animal microbiome^32, 33^, e.g. by increasing the prevalence of antibiotic resistance genes.

The comparison of domestic and feral animals should also provide information on the impact and potential deleterious effects of medication upon horse microbiome; however, in animals subjected to intense selection, it is sometimes difficult to obtain access to representative samples from wild populations. In the case of horses, there are no truly wild variants, with the exception perhaps of a small number of Przewalsky horses that inhabit remote areas of Mongolia, whose wild or feral origin is still a matter of debate^8^; however, there are a few feral populations around the world. These animals, despite belonging to breeds that were domesticated in the past, were released into the wild several decades or centuries ago, in most cases before the antibiotic era. Due to this life-pattern, the comparison of these feral animals with their domestic counterparts may help to unravel the impact of domestication upon horse microbiome.

The target of this study was to identify fecal microbiome in horses from three different continents and to assess the differences between feral and domesticated animals. The management of the animals in the domestic environment allows hypothesizing that this may affect important microbiome characteristics such as the presence of antibiotic resistance genes or the levels of genes involved in the metabolism of dietary carbohydrates. The aim was to provide a base platform of knowledge on horse microbiome that may potentially serve as a basis for the development of microbiota manipulation targets for the future modulation of horse microbiome to reduce the risk of disease.

## Results

Shotgun metagenome sequencing produced a total of 442.52 GB of sequences (average of 11.06 GB per sample) that were filtered for the downstream analysis. On average, 18.7% of the reads could be assembled into scaffolds. A total of 5,108,806 genes were predicted, which after alignment against the NR gene set allowed for generating a profile with 2,873,521 genes. To assess the robustness of our in-house method a comparison with the microbial composition profiles obtained with Metaphlan3 was conducted showing a good agreement with the data obtained using our custom pipeline (Supplementary Fig. 1), that also demonstrated a good gene assignment capability (Supplementary Fig. 2). To further evaluate our approach we used WOL and NCBI reference database for comparison observing a good agreement (Supplementary Table 1).

### Horse fecal microbiome differ based on geographical locations and habitat

**Fig. 1:**
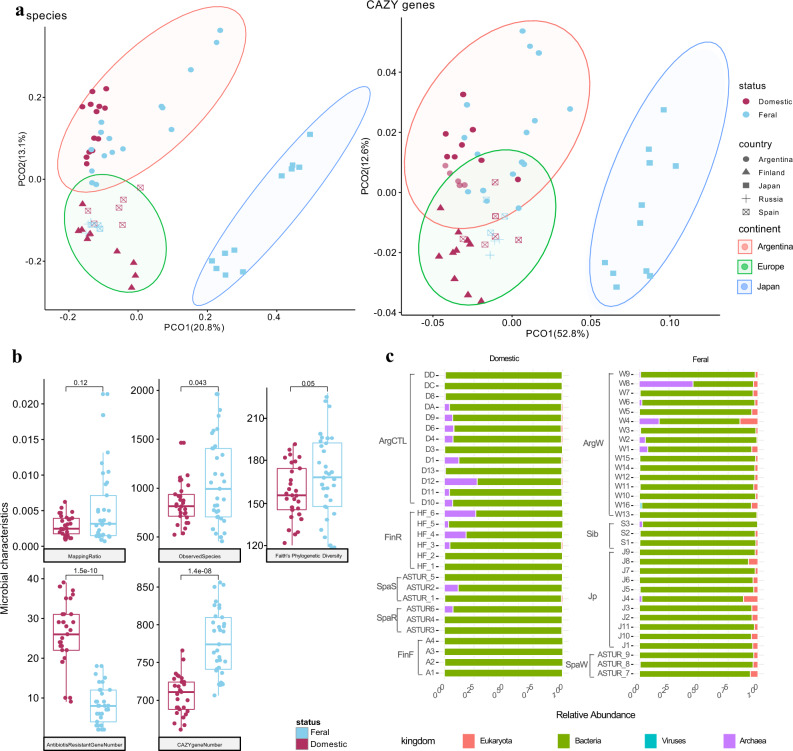
Microbial composition of feral and domestic horses from the different locations. **a** Overview of microbial composition. A principal coordinate analysis (PCOA) was performed based on the relative abundance of microbial species and the relative abundance of carbohydrate active enzymes (CAZY) with Spearman’s coefficient distance. The coloured dots represent feral or domestic samples, and the shapes indicate different cohorts. The X-axis was principal coordinate 1 (PCO1), and the y-axis was principal coordinate 2 (PCO2). The percentage values within brackets refer to the proportion of explained variance. **b** Microbial characteristics in the domestic and the feral cohorts. Including: ratio of reads with alignments with reference genomes, number of observed species, Faith’s phylogenetic diversity, number of mapped antibiotic-resistant genes and number of mapped carbohydrate-active enzyme (CAZY) families. In the boxplots the centre is the median, the whisker is 1.5 × IQR(interquartile range), and the line is the range. The boxplots show a distribution of microbial characteristics. Significance was determined by a single-sided wilcoxon rank sum test. **c** Relative abundance of taxonomy at the kingdom level. The boxplots show the distribution of the relative abundance of taxonomy at the kingdom level for all samples.

**Fig. 2:**
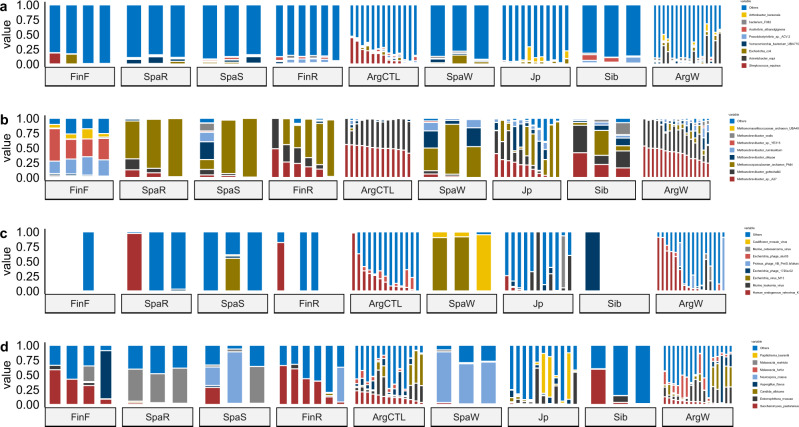
Mean relative abundance of dominant species. **a** Bacteria. **b** Archaea. **c** Virus. **d** Fungi.

Differences in the fecal microbiome depending on the geographical origin of the horses were observed, notably differentiating Japanese from Argentinian or European horses (Fig. 1a). A more detailed analysis showed that 859 species were significantly associated with geographic sites after being adjusted by feral/domestic status using the multivariate association with linear models (MaAsLin q value < 0.01) (Fig. 2, Supplementary Fig. 3, Supplementary Data 1).

The majority of the Eukaryota species found to be associated with geography were associated with fungi that might be introduced into horse gut microbiota via food, such as in the case of *Lentinula edodes* (Shiitake), which is the most popular mushroom in East Asia, and it was found to be enriched in Japanese horses. Regarding differences in bacterial species, Spanish horses were enriched in different *Lactobacillus* species, those from Finland in *Romboutsia* and the horses from Argentina in *Bifidobacterium boum*, among other microorganisms showing differences according to location (Supplementary Fig. 4).

In addition, the habitat seem to have an influence on fecal microbiome since within the same geographical location, the microbiome of the feral animals differed from that of the domestic ones. Shannon, Simpson, Chao1 or ICE diversity indexes did not differ (*p* > 0.05) between domestic and feral animals but the latter group showed significantly higher (*p* < 0.05) number of observed species and higher Gini diversity index than the domestic group (Supplementary Fig. 5).

**Fig. 3:**
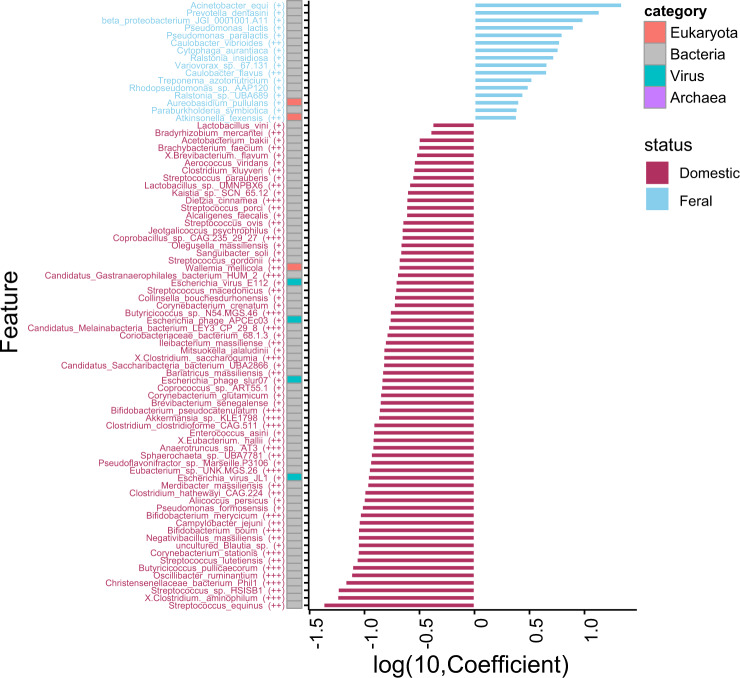
Species associated with feral or domestic environments. Statistically significant domestic/feral associated microbiota species. The X-axis is a transformed coefficient generated using Masslin2, and the coloured bars attached to the Y-axis indicate the kingdom level of species. Significance is denoted in brackets as follows: +++, qvalue < 1e-3; ++, qvalue < 0.01; +, qvalue 0.01–0.05. The coefficient transformation was determined as follows: TransCo = abs(co) * B. abs(co) is the absolute value of the coefficient, and B is 1 or −1 when the coefficient is positive or negative, respectively.

After adjustments by the different countries sixty-five and sixteen species were enriched in domestic and feral horses, respectively (Wilcoxon rank sum test, *p* value < 0.01. MaAsLin, q value < 0.05). *Acinetobacter equi* was the most differential microorganism in feral animals, whereas *Streptococcus equinus* was the species with a higher discriminating power for domestic animals (Fig. 3). Similarly, the number of sequence reads that mapped differed among the horse’s groups, underlining the existing differences in microbiome composition (Supplementary Fig. 6). These differences reached a statistical significance (*p* < 0.05) when domestic and feral animals were compared, the latter showing higher mapping values or gene richness (Fig. 1b).

The analysis of the data at the kingdom level further demonstrated the differences between domestic and feral animals (Fig. 1c, Supplementary Fig. 7), with the latter showing higher levels of genes from eukaryota (*p* < 0.05) and viruses (*p* < 0.05) and lower levels of archaea (*p* < 0.05). Bacteria in feral horses seemed to be in lower relative abundances, although the differences were not statistically significant (*p* = 0.1). Among the eukaryotes found, the yeast *Saccharomyces pastorianus* was the dominant species in domestic animals, whereas the fungi *Entomophthora muscae* was dominant in feral ones (Fig. 2). Regarding bacteria, *Streptococcus equinus* and *Acinetobacter equi* were the dominant species in domestic and feral animals, respectively. *Methanobrevibacter* was the dominant archaea in both groups of animals.

### Domestication and geographic location determine antimicrobial resistance genes (ARGs)

**Fig. 4:**
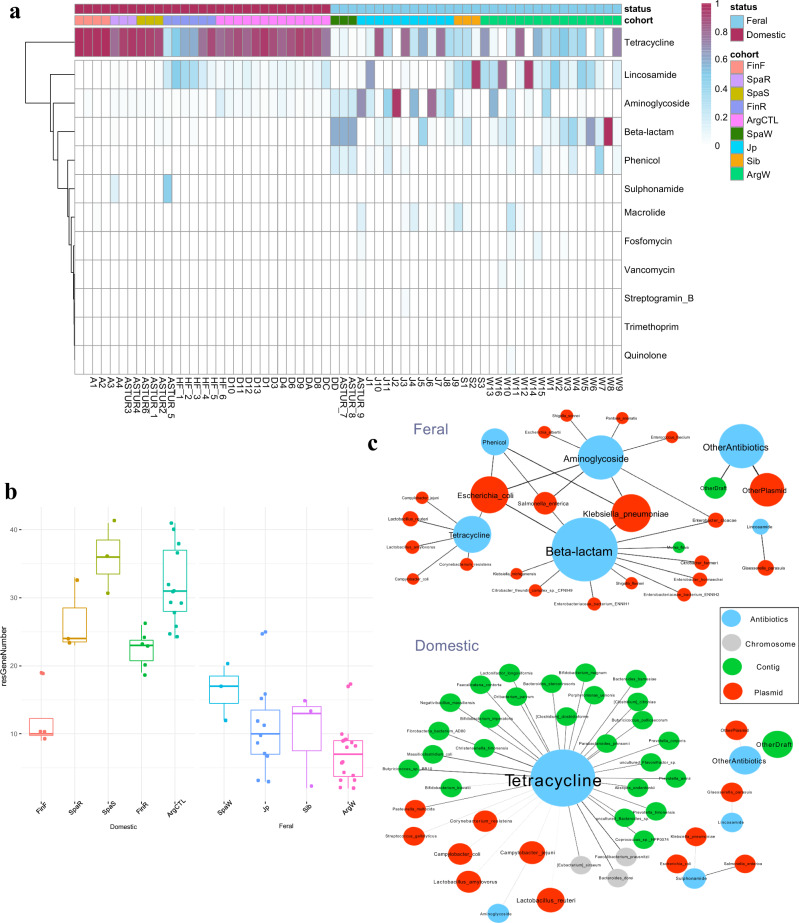
Antibiotic resistance genes in feral and domestic horses. **a** Overview of antibiotic-resistant phenotype profile. The gradient colour in the heatmap indicated a relative abundance of an antibiotic-resistant profile. The column coloured bars represent the category and the cohorts of samples. **b** Abundance of antibiotic resistance genes. The boxplots show the distribution of the abundance of antibiotic resistance genes in the different animal groups. “FinF”; Finnish farm (domestic) animals. “SpaR”; Spanish ranch (domestic) animals. “SpaS”, Spanish stable (domestic) animals. “FinR”; Finnish ranch (domestic) animals. “ArgCTL”; Angentinean stable (domestic) animals. “SpaW”; Spanish feral animals. “Jp”; Japanese feral animals. “Sib”; Siberian feral animals. “ArgW”; Argentinean feral animals. In the boxplots the centre is the median, the whisker is 1.5 × IQR(interquartile range) and the line the range. **c** Interactions of reference genomes and antibiotic-resistant genes. The node size was proportional to the mean frequency of reads assigned within the gene families of relative antibiotic-resistant phenotype or reference genomes (for source data see Supplementary Data 4 file). The line transparency is proportional to the mean frequency of reads that could be assigned to both the antibiotic-resistant phenotype and the reference genomes. The node colour indicates antibiotic-resistant phenotypes or reference genome type. Chromosome: chromosome reference genomes. Plasmid: plasmid reference genomes. Contig: contig reference genomes in which chromosomes and plasmids regions are not divided. The top and bottom panels show the feral and domestic samples, respectively.

To assess the potential impact of the habitat (domestic vs. feral) and geographical location on antimicrobial resistance, the number of ARGs was determined in both groups. The data showed differences in the levels of antimicrobial resistance among horses from different geographical origins (Fig. 4a) and a strong impact of the habitat, with domestic animals showing significantly higher (*p* < 0.05) levels of ARGs than their wild-living counterparts (Figs. 1b, 4b). It is worth noting that in domesticated horses, tetracycline resistance genes were the main group in most animals, whereas in feral animals, a larger variability was found in the dominant ARG families (Fig. 4a). Different tetracycline resistance genes, including both ribosomal protection proteins and efflux pumps, showed significantly higher levels in domestic than in feral animals (Supplementary Table 2). *tet*(Q) followed by *tet*(W) and *tet*(40) were the major ARGs found in domestic animals, whereas in feral horses, the gene *lnu*(C), encoding a lincosamide nucleotidyltransferase conferring resistance to lincomycin^34^, was the ARG found at higher levels followed by *tet*(W) (Supplementary Table 2).

The association analyses showed that in domestic animals, tetracycline resistance genes were associated with several different microorganisms, indicating that the presence of this gene is common among different species in domestic horses (Fig. 4c). On the contrary, in feral animals, the presence of tetracycline resistance genes was associated with a limited number of microbial species of *Campylobacter*, *Lactobacillus*, *Corynebacterium* and *Escherichia*. The presence of other ARGs families, such as those conferring resistance to beta-lactam antibiotics, was associated with the presence of different members of the family *Enterobacteriaceae*, whereas the resistance to lincosamide was linked to the pig pathogen *Glaesserella parasuis* (former *Haemophilus parasuis*)^35^ (Fig. 4c). Moreover, whereas in feral animals most of the ARGs seemed to be encoded into plasmids, in domestic animals, the presence of tetracycline resistance genes often seemed to have a chromosomal origin.

### Feral animals show increased levels of enzymes involved in carbohydrate metabolism

**Fig. 5:**
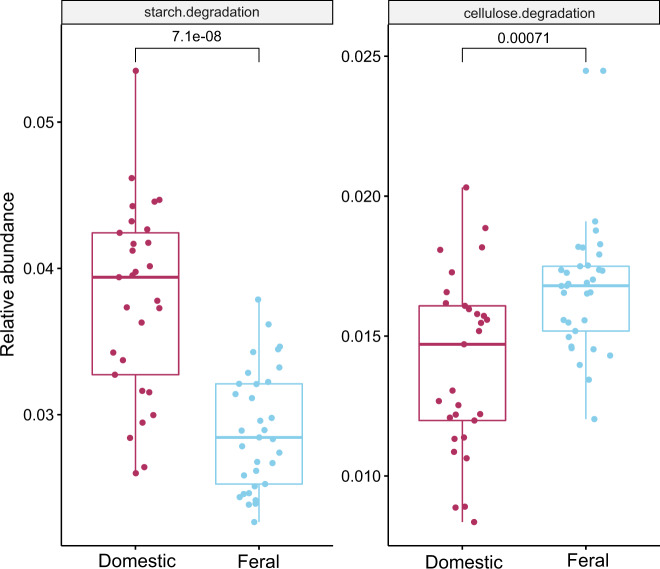
Relative abundance of cellulose and starch metabolism families. The boxplots show the distribution of the relative abundance of cellulose and starch metabolism families in the domestic and feral cohorts. Significance was determined by a one-sided Wilcoxon’s rank sum test. In the boxplots the centre is the median, the whisker is 1.5 × IQR(interquartile range) and the line is the range.

Next, the families of enzymes forming, modifying or hydrolyzing glycosidic bonds were analyzed using the CAZy database. The number of enzymes involved in the metabolism of carbohydrates was significantly higher (*p* < 0.05) in feral than in domestic animals regardless the geographic origin of the samples (Fig. 1b, Supplementary Fig. 6). This is in good agreement with the increased gene richness and diversity of these animals. The CAZy data analysis allowed for a clear differentiation among the groups of animals as shown by the PCoA analysis (Spearman correlation) (Fig. 1a). No major rearrangements were observed when the relative proportions of the different families were examined (Supplementary Fig. 8). The family glycosyltransferase (GT) 2, followed by the glycosylhydrolase (GH) 2, GT 4, GH 3, GT 35, GT 78 and GH 29, were predominant in domestic animals, whereas GT 2, GH 2, GT 4 and GH 3, followed by GH 31, GT 78 and GH 29, were dominant in feral ones. When the relative proportions of the main enzyme families were compared between domestic and feral animals, several statistically significant differences were observed (Supplementary Fig. 9, Supplementary Table 3). Among these, GT2 (including cellulose synthase, chitin synthase or N-acetylglucosaminyltransferase activities), GT4 (sucrose synthase and α-glucosyltransferase among other enzymatic activities) and GT35 (glycogen and starch phosphorylase) were significantly enriched in domestic animals. In contrast, feral animal microbiome were enriched in the families GH3 (including, among others, β-glucosidase, xylan 1,4-β-xylosidase, β-glucosylceramidase, β-N-acetylhexosaminidase and α-L-arabinofuranosidase activities) and GH31 (including α-glucosidase, α-galactosidase, α-mannosidase and other similar activities). These results are suggestive of a different plant-carbohydrate metabolism between both groups of animals. To test this, the levels of genes related to starch/glycogen metabolism and those of cellulose metabolism were calculated and compared between both groups of animals, with domestic horses having higher levels of genes related to the metabolism of starch and lower levels of those related to that of cellulose than wild-living animals (Fig. 5, Supplementary Data 2).

## Discussion

The horse gastrointestinal tract includes two large fermentation chambers, the colon and the caecum, the well-being and survival of horses rely on the undisturbed fermentative function. These chambers are inhabited by diverse microbiota, including bacteria, protozoa and fungi; however, the composition and activity of the microbiome remains largely unknown^28^. In this study, the fecal microbiota of horses from five different locations and habitats across three continents were investigated. The results show significant differences in the microbiome of horses depending on geographic origin and habitat. In spite of the large differences observed, it is important pointing out that, although the sequencing was performed in the same lab, the sample collection and DNA extraction were conducted in different laboratories, which may have included site-specific biases influencing, at least partially, the differences found.

Interestingly, domestic animals presented higher archaea levels and lower levels of eukaryotes and viruses. Therefore, domestication or captivity seems to be related to an enrichment in methanogenic species, such *as Methanobrevibacter*, and to a reduction in diversity. Although the studies in this area are scarce, Gao et al.^36^, who studied wild and captive Tibetan wild ass (*Equus kiang*), also concluded that captivity reduces diversity.

Domestication also affected the presence of ARGs, with tetracycline resistance genes becoming significantly more common in domesticated animals in all geographical locations. This common trait observed in different parts of the world suggest a key role of antibiotics in the modulation of the modern domestic horse microbiota. Tetracycline-type antibiotics are the most common antibiotic group used for horses in Europe^37^, with doxycycline being frequently used to treat infectious diseases in horses around the world^38–40^, which may facilitate the accumulation of bacteria harboring these resistance genes in domestic horses. AMR patterns of major bacteria isolated from diseased horses in France were studied between 2012 and 2016^41^. Of more than 12,000 antibiograms analyzed, proportions of resistance to tetracycline were among the highest for all the bacteria considered. Moreover, the differences on the levels of ARGs observed between northern and southern Europe countries, where data on antibiotics usage is available, shows a good correlation with antibiotics sales^37^. This suggests that the overuse of antibiotics in some countries may have affected the carriage of ARGs by the animals microbiome, underlining the importance of an appropriate use and monitoring of these drugs. It was recently reported that the common practice of widespread prophylactic antimicrobial administration in horse management practices promotes multi-drug resistance^42^. Other drugs, such as non-steroidal anti-inflammatories, have also been found to induce gut dysbiosis in horses^43^. Similarly, other pharmacological treatments used for horses’ health could potentially affect the microbiota, although the studies in this area are still scarce.

Interestingly, differences in carbohydrate metabolism enzymes were also observed between domestic and feral animals. Whereas domestic horses’ microbiome seemed to be enriched in GTs genes, feral animals presented higher levels of GHs. These differences suggest a differential metabolism of carbohydrates, with wild-living animals showing a potentially higher ability to catabolize complex carbohydrates, likely reflecting a more varied diet with a more complex and variable carbohydrate availability. In more detail, the microbiomes of feral animals are enriched in genes related to the metabolism of cellulose, whereas those of domestic ones are enriched in genes related to starch/glycogen. This is a likely reflection of the different diets, with more cereal and grains in the feeding of domestic animals, whereas wild-living ones rely on grazing and shrubby plants. This observation is in good agreement with the results of different dietary-challenge studies that demonstrated an enrichment in amylolytic bacteria and a reduction in cellulolytic microorganisms after the modification of diet by the introduction of barley^44, 45^. Moreover, the specific types of starches or celluloses consumed could also have a different impact, as has been shown for starch from different sources^46^. In addition, not just cellulose utilization gene levels may be affected since differences were also observed in the levels of methanogenic archea. In monogastric animals, such as humans, the levels of these archea, and of CH_4_ production, have been associated to the levels of H_2_ producing cellulolytic clostridia whereas in non-CH_4_ producers individual cellulose degradation seem to be related to other microorganisms^47^. These could be suggestive of a switch on microorganisms and metabolic pathways involved in cellulose degradation in feral vs. domestic horses. However, it is important to underline that, since we have not determined any functional parameter such as CH_4_ production, this is merely hypothetical.

In this work, impoverishment of the intestinal microbiota was observed in domestic horses compared to feral animals. Antibiotic exposure is one of the likely causes considering the observed increase in ARGs. This may imply health consequences because intensive sanitary and hygienic management, as may occur for domestic animals, has been associated with a lower production of IgA^48^. Moreover, common management practices of horse feeding include high-concentrate diets, low forage quality, meal feeding and confinement housing, which may have impacts on intestinal function, specifically large intestinal fermentation^49^. The larger metabolic potential regarding enzymes involved in the metabolism of carbohydrates found in feral animals likely reflects the larger complexity of their diets in open spaces compared to confined domestic animals. The importance of the available food and the environment on the horse microbiota has been demonstrated in Przewalski horses^23, 25^. Differences in gut microbiota structures were reported among horses reintroduced to the different reserves, which suggests that these findings combined with a detailed knowledge of the available and consumed food plant species could provide guidelines for the selection of potential future reintroduction sites because available natural food significantly impacted microbiome.

Taken together, this study has revealed differences in the microbiome of horses from different geographical locations. Moreover, the data revealed major differences between feral and domesticated animals, thus demonstrating the evolutionary role of domestication-associated antibiotics, feed and environment. In the future, these results could lead to novel targets for modulating microbiome and for the characterization of new combinations of horse microbes to develop effective microbiome-restoring approaches.

## Materials and Methods

### Animals and sample collection

**Fig. 6:**
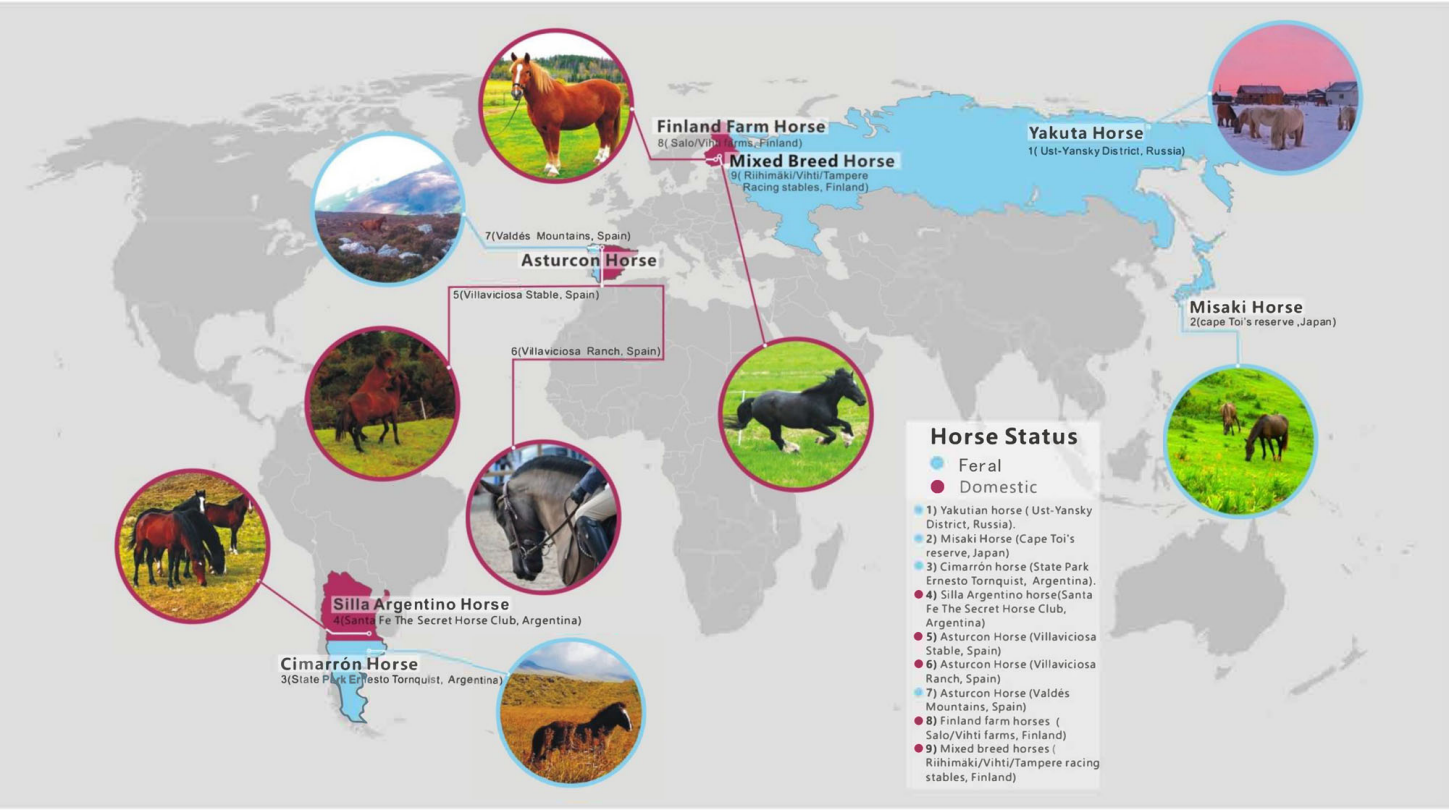
Worldwide distribution of horses. The map indicates the distribution of feral (red) and domestic (blue) horses in the study.

Fresh fecal samples were collected from 57 domesticated or feral horses. Horses were from different locations around the world (Fig. 6, Supplementary Table 4. These include Asturcon horses from Asturias (Spain), Misaki horses from Cape Toi’s Reserve (Japan), Cimarron horses from the State Park Ernesto Tornquist (Argentina), riding and competition horses from Finland and arctic Yakutian horses from the Sakha Republic (Russia). The necessary permission to sample horse feces was obtained from the corresponding authorities and/or the animal owners. Horses were reported as healthy animals by a veterinarian and/or the owner.

The samples were collected and immediately frozen in dry ice or placed under anaerobic conditions and transported under refrigeration to the lab. The transport method was chosen depending on the distance and accessibility between the sampling location and the laboratory where the samples were processed. In Argentina, Finland and Russia, the samples were immediately placed on dry ice, transported frozen to the lab and processed within 48 h. In Japan, the samples were transported to the lab under anaerobic conditions (Anaero Pack, Mitsubishi Gas Company) at 4 °C and processed within 48 h, whereas in Spain, the samples were transported under anaerobic conditions (Anaerocult A system, Merck) at 4 °C and processed within three hours from deposition.

Once in the laboratory, the samples were processed by making a 1:1 dilution in sterile 0.05-M phosphate-buffered saline (PBS; pH 7.2) in a 50-ml screw cap tube, and the samples were homogenized and centrifuged at 200 × *g* for 5 min to sediment the residual coarse particles and grass. The supernatant was then transferred to a new sterile tub, vortexed and centrifuged at 9000 × *g* for 4 min. The pellet was washed with PBS, centrifuged again and then used for DNA extraction with the commercial kit QIAamp DNA Stool Mini Kit (Qiagen, Hilden, Germany).

### Microbiome analyses

#### DNA sequencing

Extracted DNA was precipitated by standard ethanol/sodium acetate precipitation and delivered to the Gene Hospital Department from The First Affiliated Hospital of Zhengzhou University at room temperature within one week. DNA degradation degree and potential contamination were monitored on 1% agarose gels. The concentration was measured using the cubit® dsDNA Assay Kit in Qubit® 2.0 Flurometer (Life Technologies, CA, USA). DNA was randomly sheared with Covaris, and the DNA library was then constructed using the NEBNext UltraTM DNA Library Prep Kit for Illumina (NEB, USA) according to the manufacturer’s instructions. The DBNA library was sequenced using Illumina NovaSeq 6000 with a pair-end 150 bp sequencing strategy.

#### Sequence processing

Quality control (QC) was performed with a custom script. Sequences were discarded if one of the following criteria were met: (1) Contained two or more ambiguous bases; (2) contained 30 or more low Phred quality bases (threshold, Q20); (3) were assigned as adapter sequences using Cutadapt version 1.8.1 with ‘-u 6’ parameter^50^; (4) could be aligned against *Arabidopsis thaliana* (NCBI RefSeq assembly accession: GCF_000001735.3) or *Equus caballus* (NCBI RefSeq assembly accession: GCF_000002305.2) using bowtie2 version 2.3.4.2^51^ with ‘—very-sensitive’ parameter; or 5) no more than 29 low-quality bases at the 3′ end were trimmed out (the whole sequence was still kept). For pair-end sequences, if one sequence was considered an adapter contamination, *A. thaliana* or *E. caballus*, its mate sequence was also filtered out. Finally, the sequences that passed QC were used for the downstream analysis.

#### Reference genome database construction

We built our prokaryotic reference genome based on NCBI prokayotic genomes as of March 15, 2018 with the following method: (1) Complete genomes and draft genomes were processed separately and then combined. (2) Within one species, we calculated the genome length and discarded genomes with abnormal length and GC content (Tukey’s Fences with custom coefficient as 2, these genomes with abnormal genome length and GC content might be with contamination or with missing regions). Then we chose three genomes with longest genome length. (3) for genomes which do not have a specific scientific name at species level (for example, Erythrobacter sp. NAP1) we pooled these genomes together according to their genus level and make a single complete clustering method with distance threshold 0.9. Finally we chose the longest genome within one cluster as representative genome. All fungi and virus genome were downloaded on March 15, 2018, and kept (Supplementary Data 3).

#### Microbial abundance profiles

Alignments from 15 million (4.5 giga bases) randomly chosen pair end reads were chosen from each sample. Bowtie2 aligner v2.3.4.2 was used to align reads against reference genome and non-redundant gene set with default parameter except “-a” (report all qualified alignments), then candidate profile was then constructed with algorithm introduced by Li^52^, microbial species detected with rare frequency (no more than 2 samples) were filtered out. For one sample, to solve the randomicity of sampling reads, we repeat this process 100 times, then we will get 100 species profiles (matrix format table) and we use mean value of these matrix for final species profile. To check the in-house method used at the time of analyses, using the UNICO algorithm^53^ a brief comparison was done with the more recently developed Metaphlan3. To this end the Metaphlan database version mpa_v30_CHOCOPhlAn_201901, was used to generate microbial composition data to compare with our custom approach data.

#### CAZy functional profile

The pair end sequences were aligned against the curated dbCAN2 database (version is CAZyDB.07312018.fa)^54^ by using DIAMOND protein aligner version 0.9.24^55^ with the parameter ‘diamond blastx -f 6 –sensitive’ respectively. The DIAMOND aligner could only align single reads so mated reads were aligned separately, and then the alignments were merged with score more than 60 and E value less than 1e-5. If one read have qualified alignments with several genes, the abundance of this read (total abundance is 1) will be divided by number of mapped genes. For each sample, frequency of alignments could be assigned with the same CAZy genes were added as functional abundance and adjusted by related gene length.

#### Antibiotics resistant profile

The pair end sequences were aligned against CARD resistomes database (version 3.0.1)^56^. The alignments were used to calculate the interaction between antibiotic-resistant phenotypes and reference genomes. For each sample, if one read could be assigned to both an antibiotic-resistant phenotype and a reference genome, then this reference genome could contribute to the specific antibiotic-resistant phenotype. Finally, the mean frequency in all samples in each cohort (domestic and feral) was used as interactions and integrated and visualized by using Cytoscape version 3.7.2.

### Statistics and reproducibility

Statistical analyses were performed using R version 3.6.1. The wilcoxon rank sum test was performed to compare the microbial characteristics, microbial species and function composition. The corresponding *p* < 0.05 was considered statistically significant. MaAsLin2^57^ was used to find species significantly associated with geographic sites or domestic/wild environments with default parameters.

### Reporting summary

Further information on research design is available in the Nature Research Reporting Summary linked to this article.

## Supplementary information

Supplementary Information

Description of Additional Supplementary Files

Supplementary data 1

Supplementary data 2

Supplementary data 3

Supplementary data 4

Reporting Summary

## Data Availability

Sequences are available at the European Nucleotide Archive PRJEB39149. Raw data used for the Figures shown in the manuscript are available in Supplementary Data 4 file.

## Code availability

Source code, data for analysis and visualization are available at 10.5281/zenodo.5873410.
