# Supplementary material for: Gut Microbiome Characteristics in feral and domesticated horses from different geographic locations
Source: Commun Biol. 2022 Feb 25;5:172. doi: 10.1038/s42003-022-03116-2 (PMC8881449; doi:10.1038/s42003-022-03116-2)
Supplement: Supplementary file 1 — Supplementary Information [file 42003_2022_3116_MOESM1_ESM.pdf]

SUPPLEMENTARY FILE: FIGURES AND TABLES

Gut Microbiome Characteristics in feral and domesticated horses from different geographic locations

Li Ang[1,2,3], Gabriel Vinderola[4], Akihito Endo[5], Juha Kantanen[6], Chen Jingfeng[1], Ana Binetti[4], Patricia Burns[4], Shi Qingmiao[2,3], Ding Suying[1], Yu Zujiang[2,3], David Rios-Covian[7], Anastasia Mantziari[8], Shea Beasley[8], Carlos Gomez-Gallego[8,9], Miguel Gueimonde[7,*], Seppo Salminen[8,*]

[1] Health Management Centre, The First Affiliated Hospital of Zhengzhou University, Zhengzhou, China. [2] Department of Henan Gene Hospital, The First Affiliated Hospital of Zhengzhou University, Zhengzhou, China. [3] Department of Infection Disease, The First Affiliated Hospital of Zhengzhou University, Zhengzhou, China. [4] Instituto de Lactología Industrial (INLAIN, UNL-CONICET), Facultad de Ingeniería Química, Universidad Nacional del Litoral, Santa Fe, Argentina. [5] Department of Food, Aroma and Cosmetic Chemistry, Tokyo University of Agriculture, Hokkaido, Japan. [6] Production Systems, Natural Resources Institute Finland, Jokioinen, Finland. [7] Department and Microbiology and Biochemistry of Dairy Products. Instituto de Productos Lácteos de Asturias (IPLA-CSIC). Asturias, Spain. [8] Functional Foods Forum, Faculty of Medicine, University of Turku, Turku, Finland. [9] Institute of Public Health and Nutrition, University of Eastern Finland, Kuopio, Finland.

* *Joined corresponding authors*:

* Miguel Gueimonde. IPLA-CSIC, Paseo Rio Linares s/n, 33300 Villaviciosa, Asturias, Spain. Email: mgueimonde@ipla.csic.es. Orcid: htpps://ORCID.ORG/0000-0002-0192-901X.
* Seppo Salminen. Functional Foods Forum, PharmaCity, Itäinen Pitkäkatu 4 A, 5th floor. FI-20014,Turku, Finland. Email: seppo.salminen@utu.fi. Orcid: htpps://ORCID.ORG/0000-0002-9337-7642

Supplementary Figures

Supplementary Figure 1. a. Comparison of metaphlan3 and custom pipeline (Top panel, species not detected (removed) and newly found (metaphlan3_only) in all samples. Bottom panel, distribution of spearman coefficients and p values (54 out of 59 comparisons showed significantly consistent distribution, p < 0.05). **b.** Schematic diagram of UNICO algorithm (for further details see ref 53).

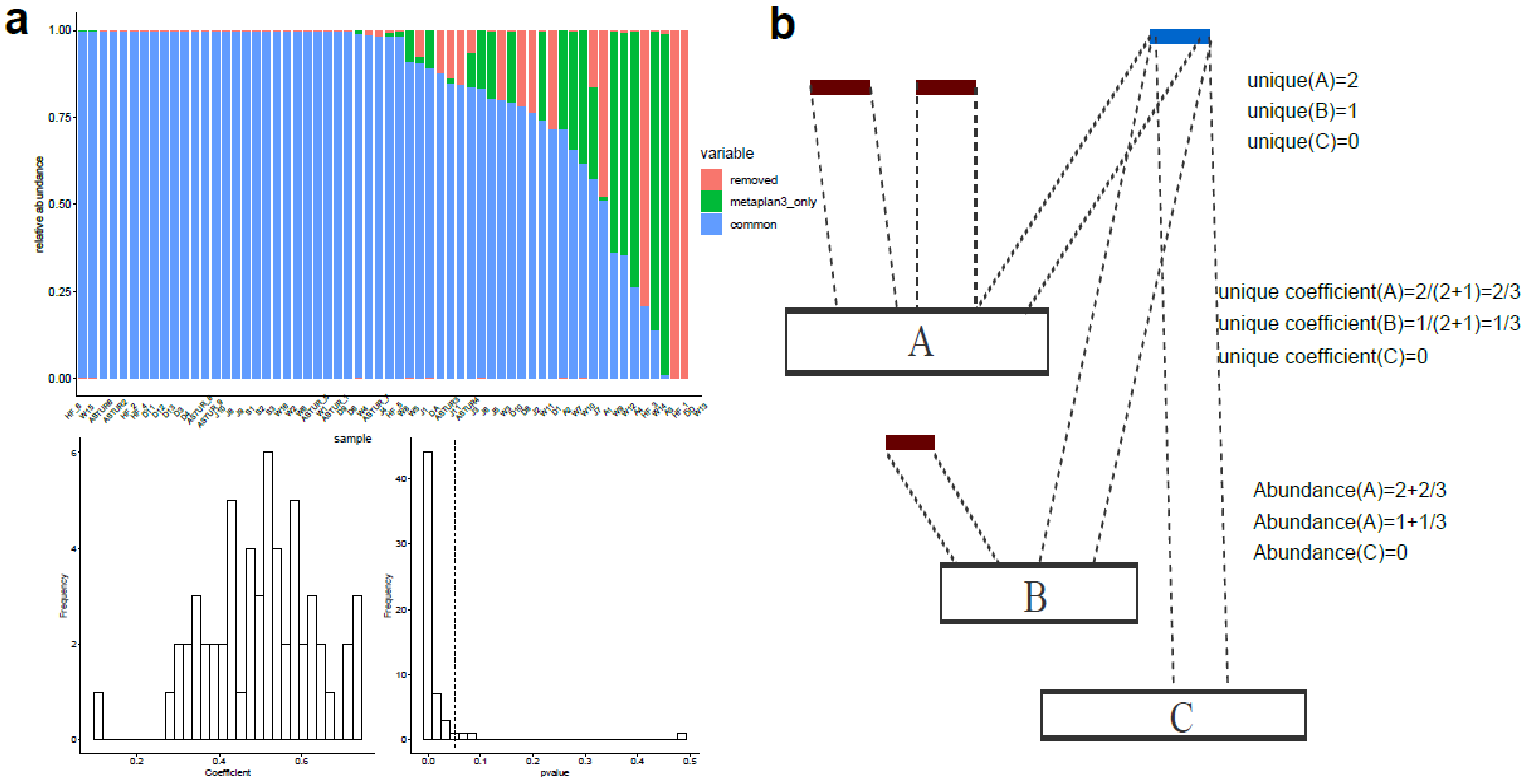

Supplementary Figure 2. Assignment of the genes into the different gene categories with the custom pipeline used in this study. Distribution of categories. Unique: reads into just one category. Multiple: reads could be assigned to multiple categories. Cross: reference genes could be assigned to different categories, but there is a common category among all genes.

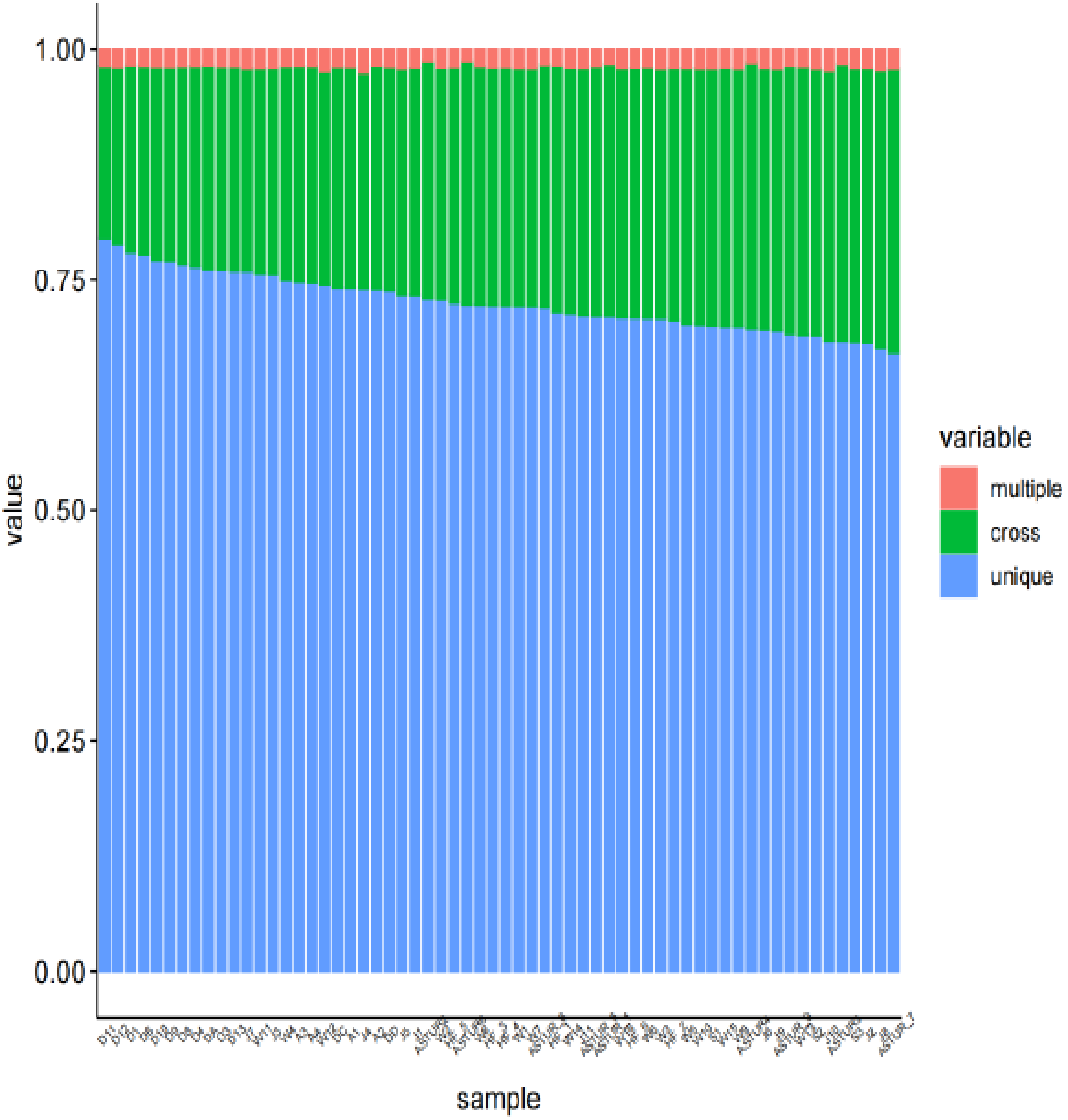

Supplementary Figure 3. Species associated with geographic sites. The heatmap shows statistically significant correlations between geographic sites and microbiota. The gradient colour in the heatmap indicates the relative abundance of microbiota. The column coloured bars represent domestic/feral status and geographic sites of all samples. The row coloured bars represent the taxonomy at the kingdom level and the microbiota enrichment. The row dendrogram tree was clustered using the complete linkage method and Euclidean distance.

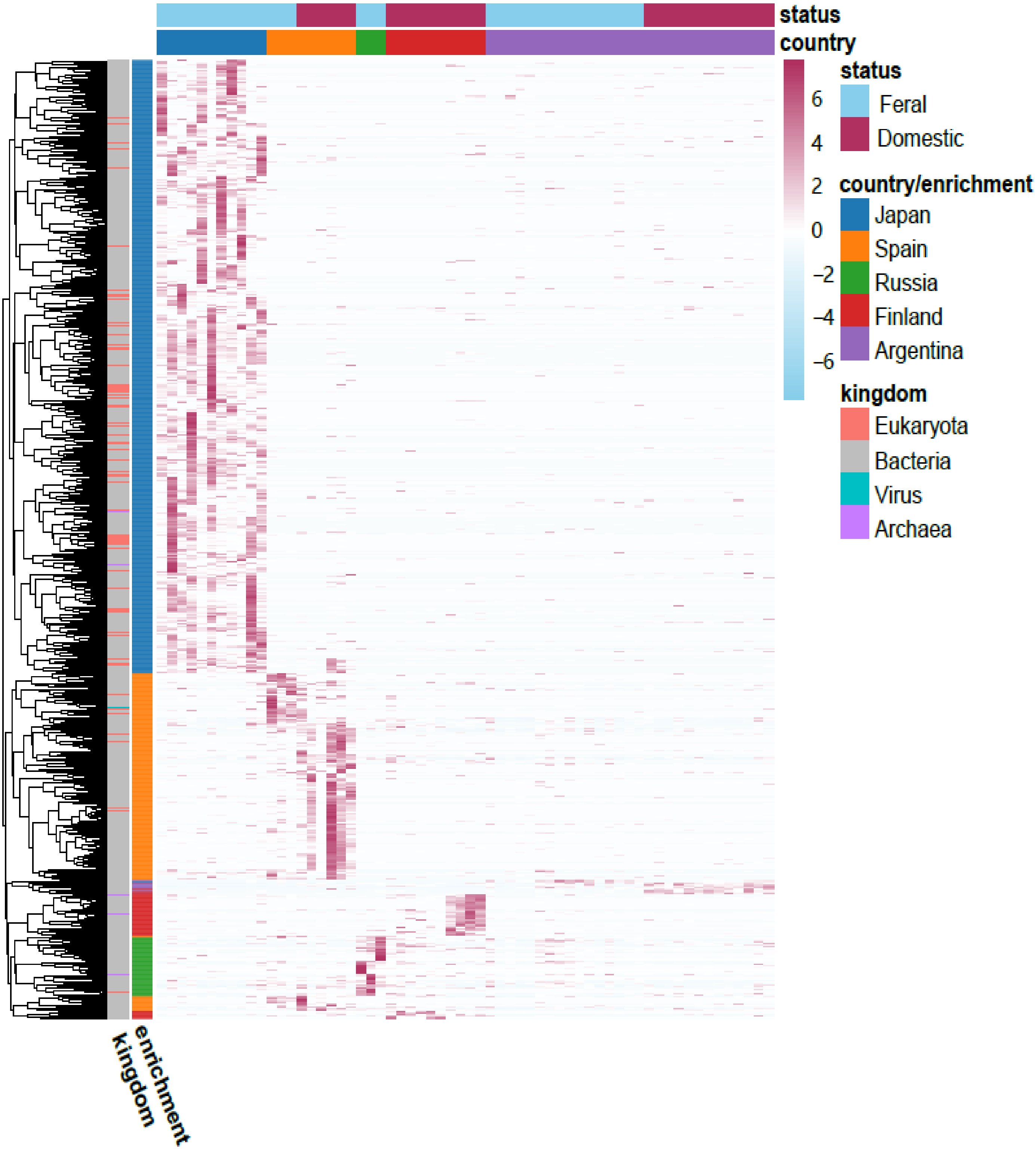

Supplementary Figure 4. Main microorganisms characteristic on the microbiome of the horses from the different locations. In the boxplots the centre is the median, the whisker is 1.5×IQR(interquartile range) and the line the range.

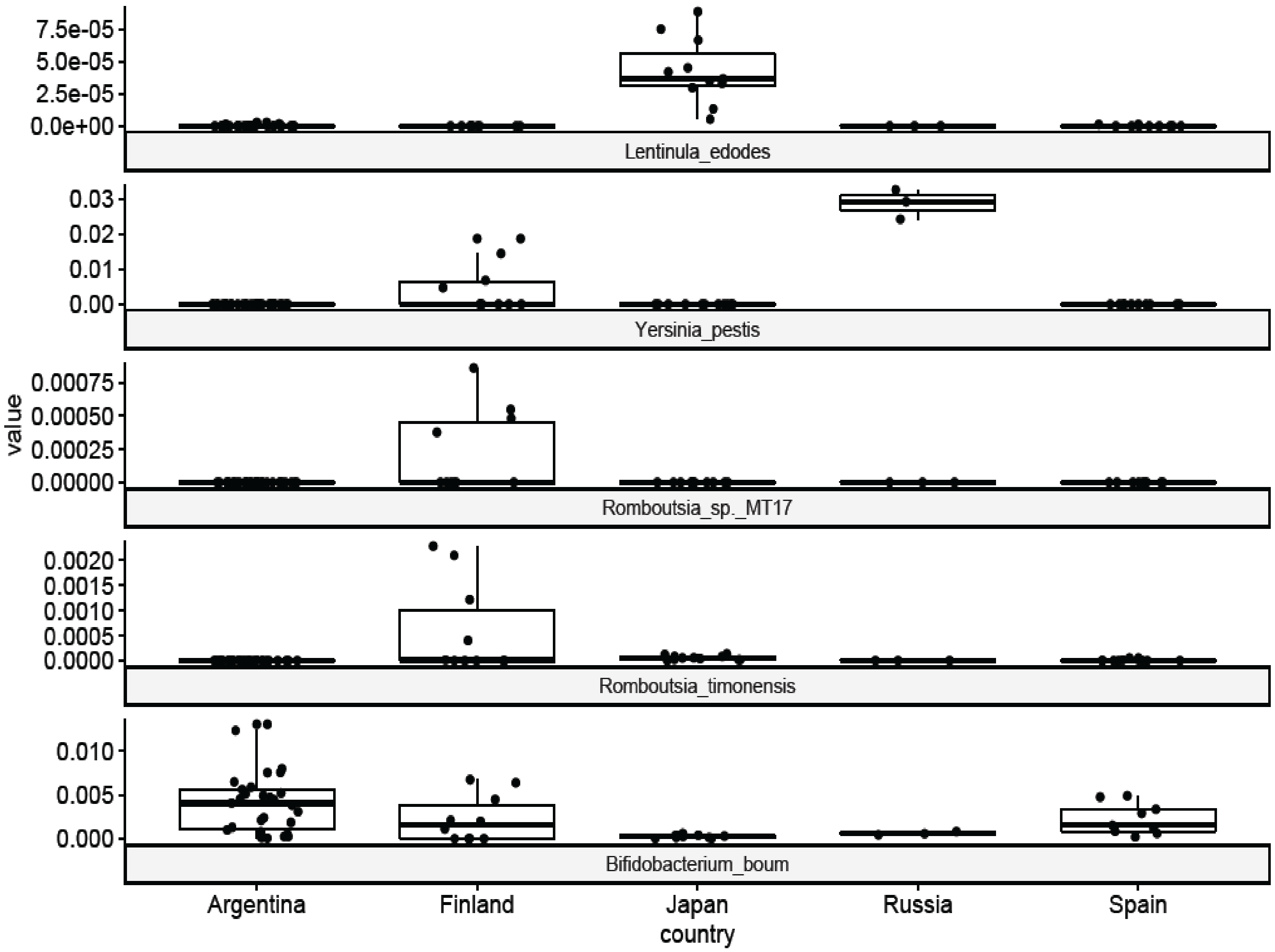

Supplementary Figure 5. Boxplots (median, 1.5×IQR and range) obtained for the number of observed species and the diversity indexes Shannon, Simpson, Gini, Chao-1 and ICE, in both domestic (pink plots) and feral (blue plots) groups. Significance was determined by a single-sided Wilcoxon rank sum test.

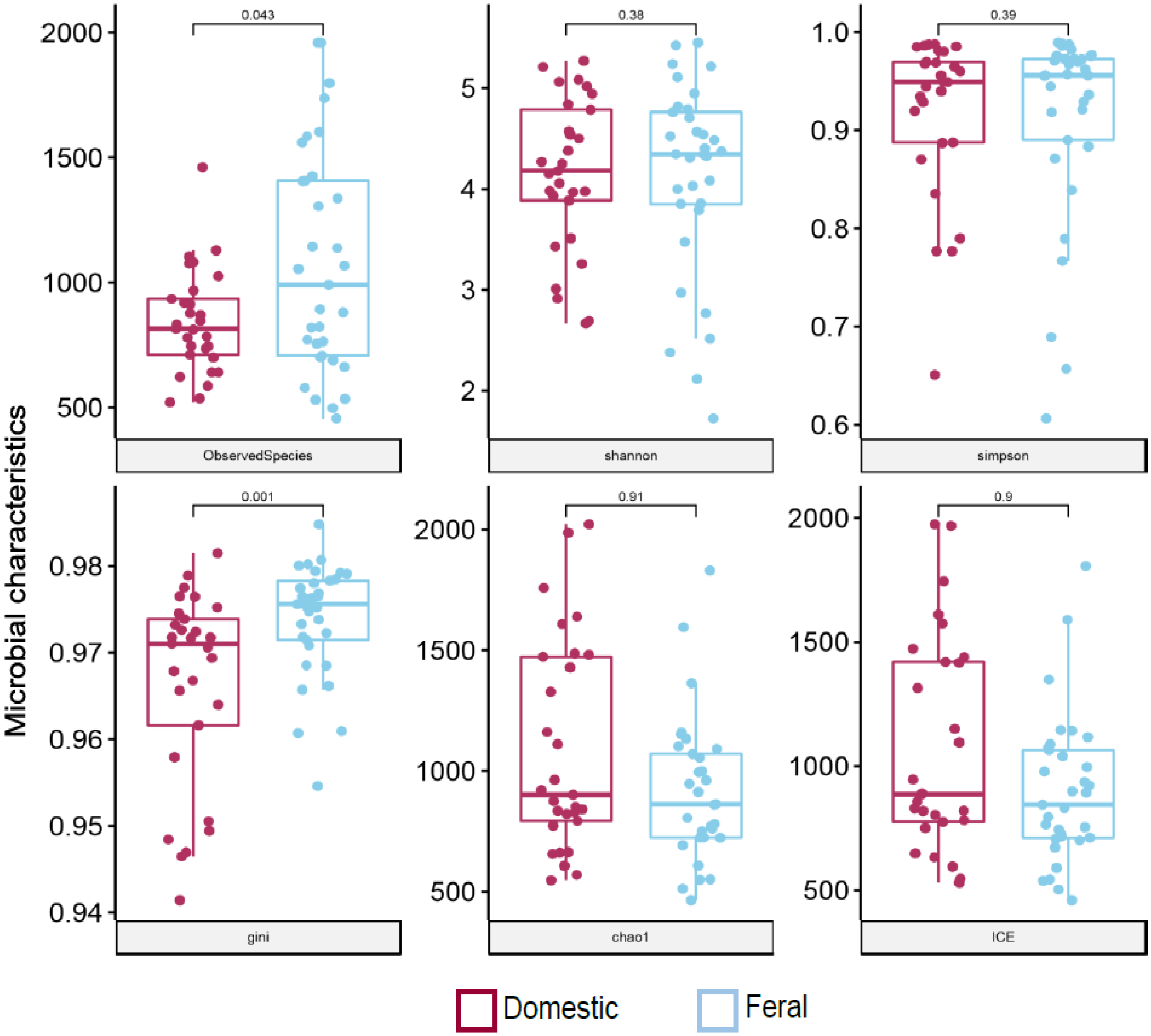

Supplementary Figure 6. Microbial characteristics in all samples. **a,** Ratio of reads with alignments with reference genomes. **b,** Number of mapped antibiotics resistant genes. **c,** Number of mapped carbohydrate-active enzymes (CAZY) families. In the boxplots the centre is the median, the whisker is 1.5×IQR(interquartile range) and the line is the range.

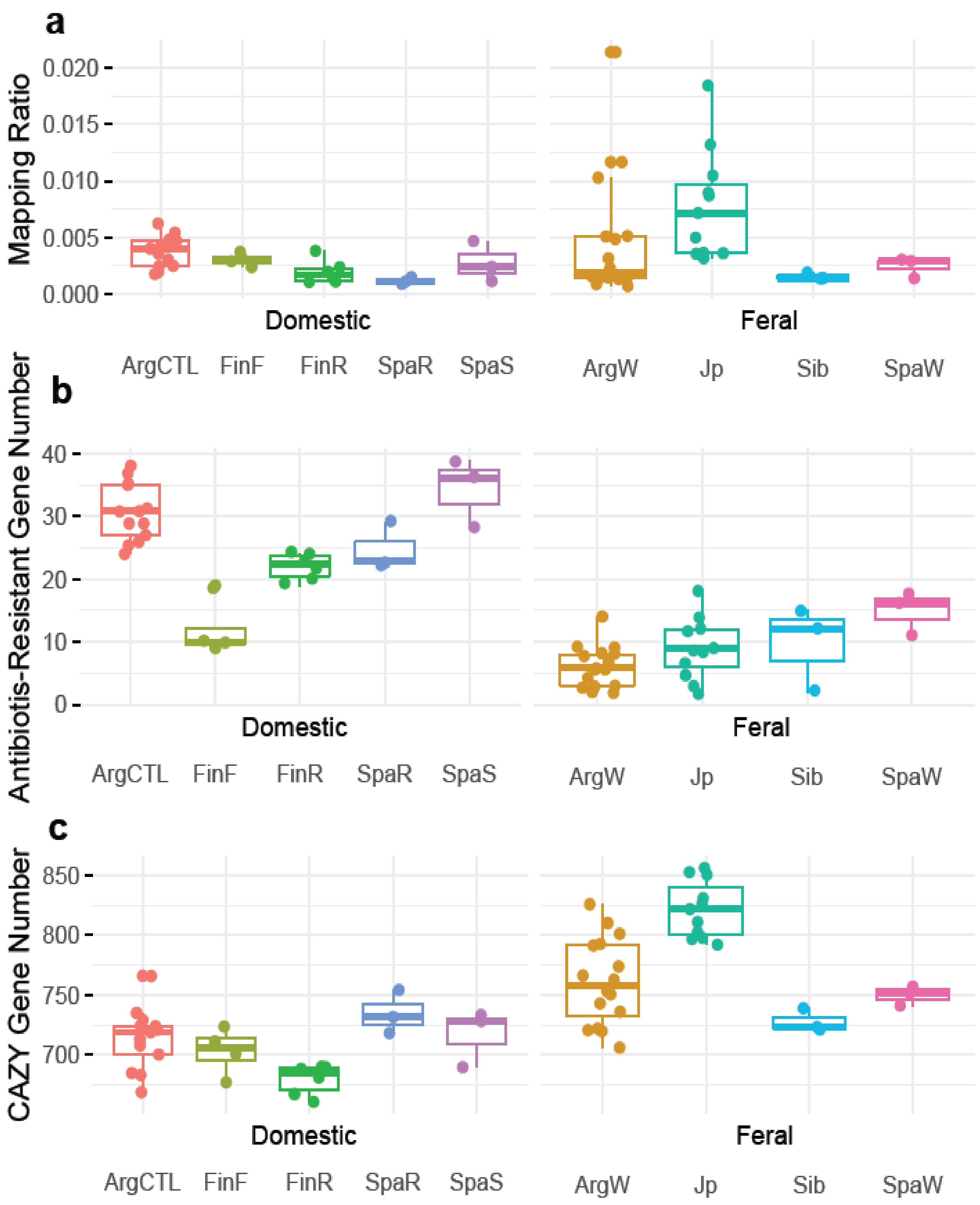

Supplementary Figure 7. Relative abundance of taxonomy at kingdom level. Boxplots showed distribution of relative abundance of taxonomy at kingdom level in domestic and wild cohort. Significance determined by one-sided wilcoxon's rank sum test. In the boxplots the centre is the median, the whisker is 1.5×IQR(interquartile range) and the line is the range.

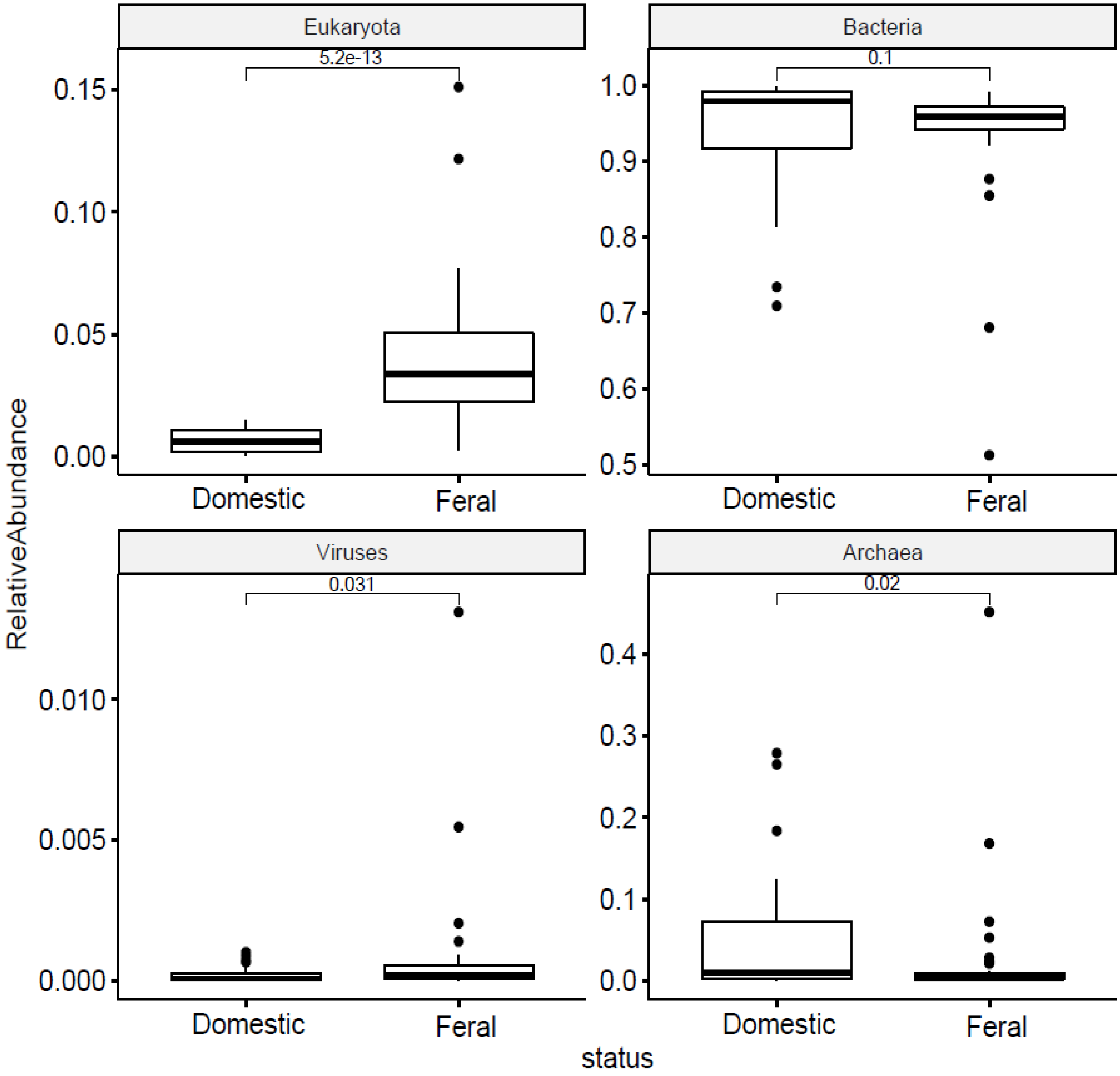

Supplementary Figure 8. Relative abundance of dominant carbohydrate-active enzymes (CAZY) families. **a,** Mean relative abundance of top 15 CAZY families in different cohorts. **b,** Relative abundance of top 15 CAZY families in all samples.

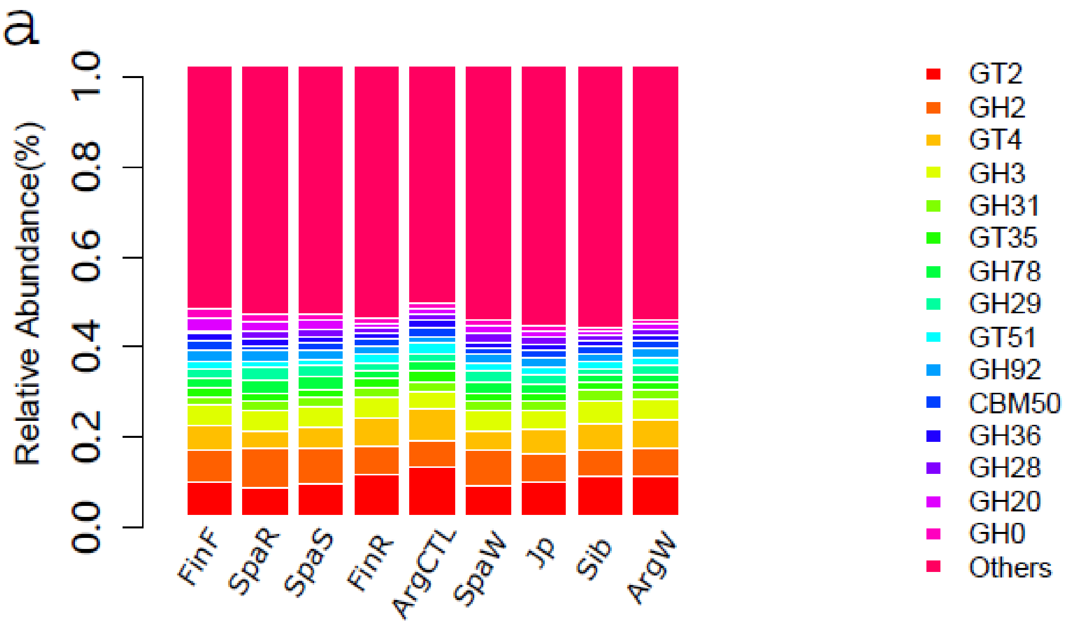

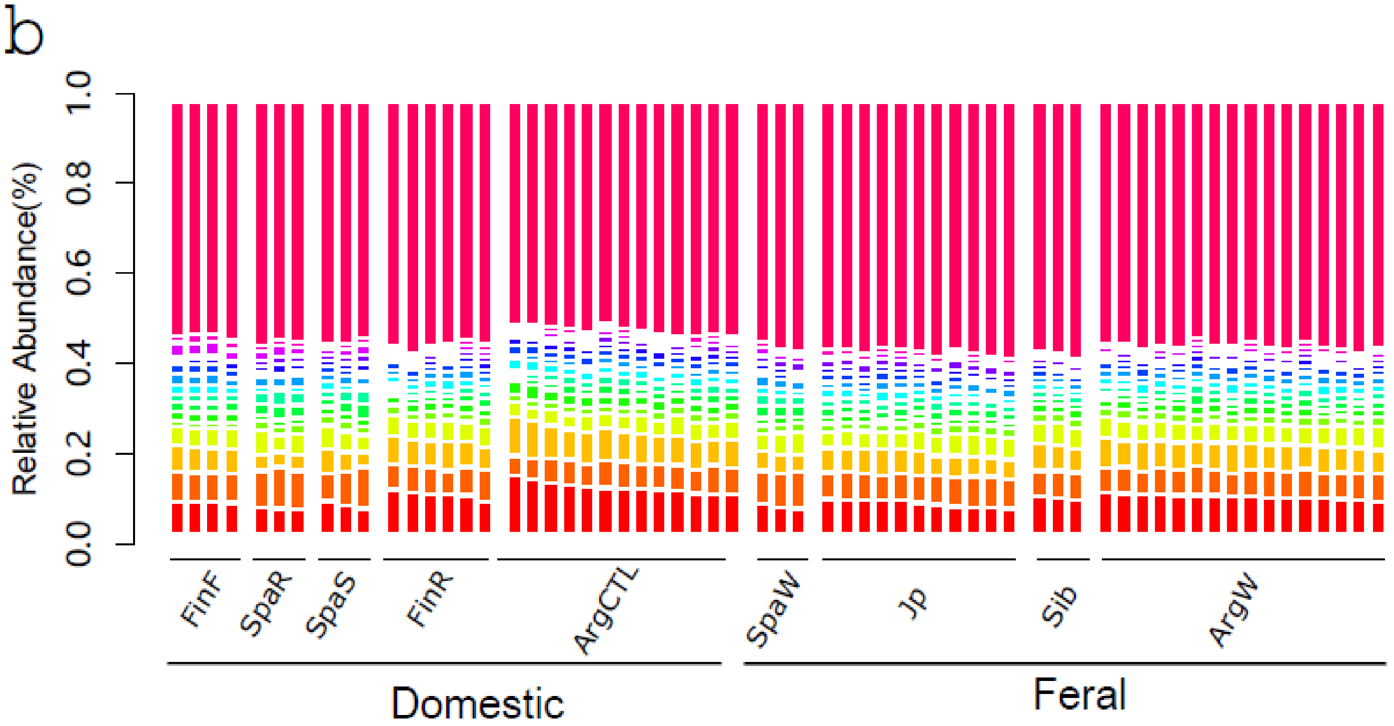

Supplementary Figure 9. Relative abundance of carbohydrate-active enzymes (CAZY) families. Significance determined by one-sided wilcoxon's rank sum test. Boxplots showed distribution of Relative abundance of carbohydrate-active enzymes (CAZY) families in domestic and feral cohort. GT, glycosyltransferases. CE, carbohydrate esterases. GH, glycoside hydrolases. CBM, carbohydrate-binding modules. PL, polysaccharide lyases. AA, auxiliary activities. In the boxplots the centre is the median, the whisker is 1.5×IQR(interquartile range) and the line the range.

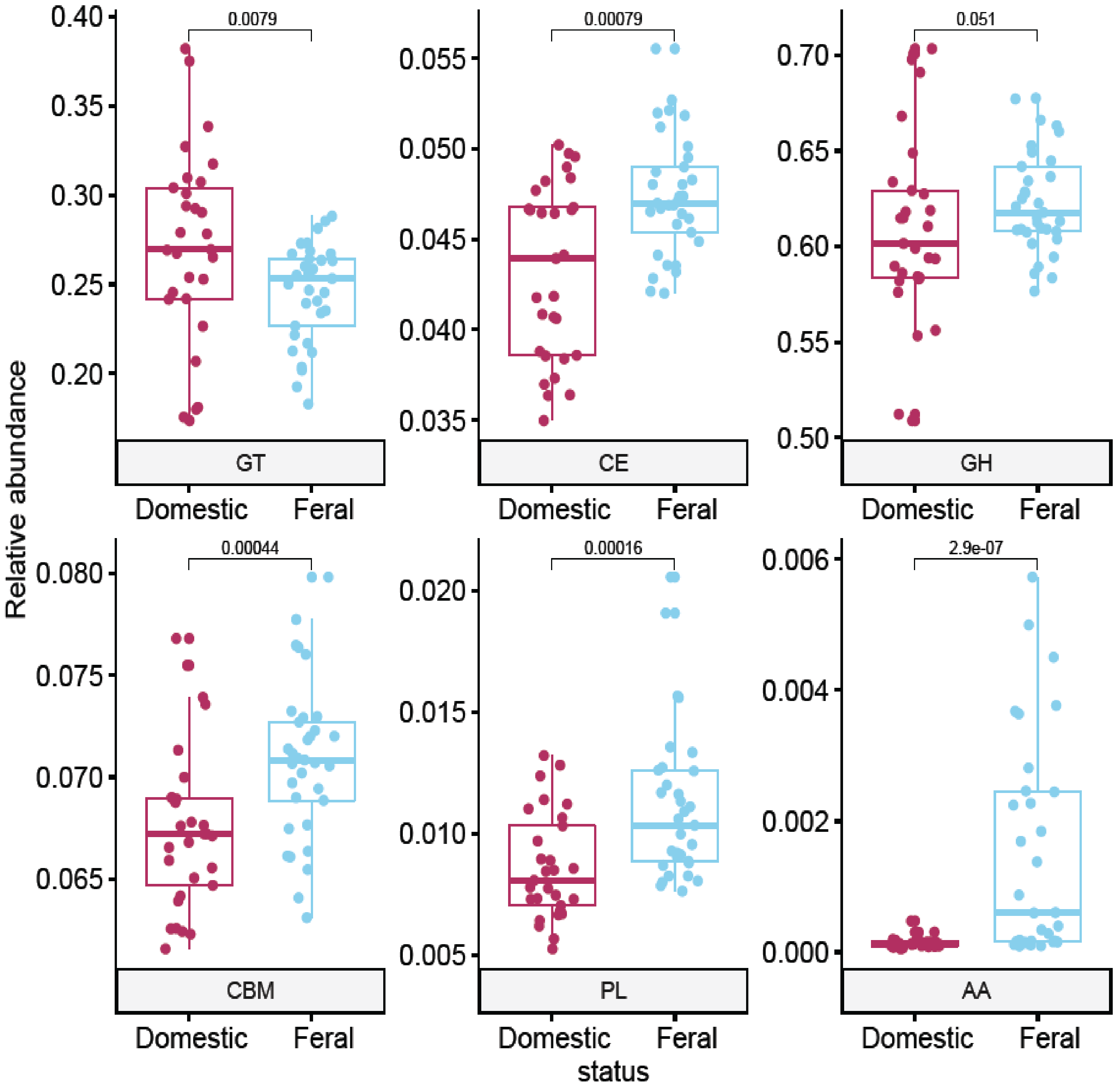

Supplementary Tables

Supplementary Table 1. Summary of species obtained in WOL, NCBI and custom pipeline.

Database	Number of genomes (NCBI GCA index)	Number of archived species	Number of species with qualified alignments [c]
WOL	10569 [a]	9100	3278
NCBI Reference (date 20210811)	13818	13800	5862
Custom Reference [b]	20728	17309	5031

[a] Seven WOL genomes were obsolete and did not have valid FTP pathways.
[b] Without Virus and Fungi genomes.
[c] Species detected in no more than 2 samples were discarded.

Supplementary Table 2. Most abundant gene resistance genes found in domestic and feral horses (group average). Significance determined by one-sided wilcoxon's rank sum test.

Gene	Domestic	Feral	*p*
tet(W)	0.222939665532665	0.113019294699326	7.38E-07
lnu(C)	0.10993322440535	0.230537179464704	0.230001405
tet(Q)	0.27110662648762	0.0377976648700862	4.55E-07
tet(O)	0.101954502644966	0.0458426590894871	2.31E-08
tet(40)	0.167336084202892	0.0458636774136819	4.13E-05
aph(2")-If	0.000243341076517981	0.0967251445302725	0.21658908
aph(3')-IIa	0.00107308505758425	0.0487703852233461	0.014930149
tet(32)	0.0485568575052838	0.00596468953566234	8.11E-10
*bla*OXA-60	0	0.0556843498268327	0.027240479
ant(6)-Ia	0.0189307364777738	0.023930602408381	8.10E-07
*bla*OXA-22	0	0.0387199678735602	0.000378016
*cat*A1	0.000571852556248481	0.0216314151835446	0.003507446
*sul*2	0.0380821592051364	0	0.067083876
Others	0.0104797656413996	0.188687786711579	4.47E-06

Supplementary Table 3. Main glycosyltranstferase and glycosylhydrolase families showing statistically significant differences between domestic and feral horses. Significance determined by one-sided wilcoxon's rank sum test.

Family		Enriched	*P* (domestic vs feral)
Glysosyltransferases	*GT2*	domestic	0.0085
	GT4	domestic	0.0187
	GT35	domestic	0.0000
Glysosylhydrolases	*GH3*	feral	0.0046
	GH31	feral	0.0125

Supplementary Table 4. General description of the animals included in the study.

Country	Animal setting		Number of animals	Main diet	Location (GPS)
Argentina	Domestic	Competition	12	Dry alfalfa and oat/feed.<br>Tab water	31.26.03 S<br>60.56.54 W
	Feral		12	Grazing<br>Natural water	38.05.14 S<br>61.56.37 W
Finland	Domestic	Farm	4		60.21.89 N<br>22.22.71 E
		Competition	6		
Japan	Feral		11	Grazing<br>Natural water	31.22.03 N<br>131.20.45 E
Russia	Feral		3	Grass<br>Natural water	71.10.42 N<br>136.28.29 E
Spain	Domestic	Farm	3	Grazing/dry hay<br>Tab water	43.30.90 N<br>5.26.34 W
		Competition	3	Grazing/dry hay/feed<br>Tab water	43.30.90 N<br>5.26.34 W
	Feral		3	Grazing<br>Natural water	43.27.33 N<br>06.36.75 W
