# Supplementary material for: Gut Microbiome Characteristics in feral and domesticated horses from different geographic locations
Source: Commun Biol. 2022 Feb 25;5:172. doi: 10.1038/s42003-022-03116-2 (PMC8881449; doi:10.1038/s42003-022-03116-2)
Supplement: Supplementary file 2 — Description of Additional Supplementary Files [file 42003_2022_3116_MOESM2_ESM.pdf]

Description of Additional Supplementary Files

File name: Supplementary Data 1

Description: Species associated with geographical sites on the studied horses.

File name: Supplementary Data 2

Description: Starch and cellulose degradation genes differing between feral and domestic animals.

File name: Supplementary Data 3

Description: Reference genomes used in this study.

File name: Supplementary Data 4

Description: Raw data used for the manuscript figures.
